# Changes in health care utilisation following a reform involving choice and privatisation in Swedish primary care: a five-year follow-up of GP-visits

**DOI:** 10.1186/1472-6963-13-452

**Published:** 2013-10-31

**Authors:** Anders Beckman, Anders Anell

**Affiliations:** 1 Faculty of medicine, Lund University, IKVM Allmänmedicin, Jan Waldenströmsgata 35, Skånes universitetssjukhus, Malmö SE-205 02, Sweden; 2 Institute of Economic Research, Lund University School of Economics and Management, P.O. Box 7080, Lund SE- 220 07, Sweden

**Keywords:** Primary care, Health care utilisation, Equity, Choice, Privatisation, Sweden

## Abstract

**Background:**

The organisation of Swedish primary health care has changed following introduction of free choice of provider for the population in combination with freedom of establishment for private primary care providers. Our aim was to investigate changes in individual health care utilisation following choice and privatisation in Swedish primary care from an equity perspective, in subgroups defined by age, gender and family income.

**Methods:**

The study is based on register data years 2007 – 2011 from the Skåne Regional Council (population 1.2 million) regarding individual health care utilisation in the form of visits to general practitioner (GP). Health utilisation data was matched with data about individual’s age, gender and family income provided by Statistics Sweden. Multilevel, logistic regression models were constructed to analyse changes in health utilisation in different subgroups and the probability of a GP-visit before and after reform.

**Results:**

Health care utilisation in terms of both number of individuals that had visited a GP and number of GP-visits per capita increased in all defined subgroups, but to a varying degree. Multilevel logistic regression showed that individuals of both genders aged above 64 and belonging to a family with an income above median had more advantage of the reform, OR 1.25-1.29.

**Conclusions:**

Reforms involving choice and privatisation in Swedish primary health care improved access to GP-visits generally, but more so for individuals belonging to a family with income above the median.

## Background

Primary health care plays a central role in most health care systems and strengthening primary care is widely seen as central in enhancing equity and efficiency in health care [1-3]. In Sweden, health care is largely publicly funded and provided and the responsibility of 21 county councils. An inhabitant makes roughly three visits to a physician per year, half with specialists in family medicine and half with other specialists, a low figure compared to other countries. About one third of practices in primary care are privately owned, but with large differences between county councils who fund and regulate their number and activities. There is no formal gate-keeping function of primary care but in order to steer demand, different patient fees for specialists in family medicine and other specialists have been instigated [4].

A wave of primary care reforms involving choice for the population and privatisation of providers was initiated across county councils starting in 2007. Important objectives behind reforms were to strengthen the role of primary health care in general and to improve performance in terms of access and responsiveness to patient expectations [5]. Choice of provider and freedom of establishment for private primary care providers became mandatory for county councils in 2010 through a change in the national Health Care Act [6]. Still, individual county councils determine the specific mechanisms of the reform and requirements on practices. Universally, individuals are required to register with a private or public practice (not with the individual family physician) without geographical restrictions and without the possibility for practices to deny registration. Choice by individuals is primarily passive on the grounds of prior use or proximity, but with an option to change provider actively [7]. Payments to practices are the same irrespective of ownership and largely determined by capitation adjusted for individual age or illness (diagnoses) and socioeconomic indicators.

Patient choice of provider is in theory expected to improve efficiency, quality and responsiveness of the health care system through the threat of exit [8,9]. As patients can exit from their current relationship if they are not satisfied, they can cause a loss of income for the providers [10,11]. A number of requirements is however necessary to achieve a positive outcome. First of all individuals must have an interest in choice and they must be well informed about the alternatives and their quality attributes. Second, individuals must have alternative providers to choose from. Causal factors for the utilisation of health care are also heterogenic, and need or health-status is just one of these factors [12]. Other important factors which can be attributed to the individual include age, gender, health literacy and socioeconomic status [13,14]. Furthermore, utilisation of health care depends on the availability of supply, which depends on geographical location, provider’s waiting-times and opening hours, presence of a gate-keeping function etc. Since utilisation of health care is complex and determined by several factors, a structural change might affect utilisation behaviour in unexpected ways, depending on available information and health literacy [14,15]. Furthermore, the ability to exercise choice is affected by income and/or education [11], so that inequalities might increase. In case individual’s interest, health literacy and/or access to alternatives differ, the benefits created by choice and privatisation may therefore vary across subgroups defined by age, gender and socioeconomic status. The main argument against choice of provider has not surprisingly been that such reforms create unequal benefits depending on socioeconomic status [16].

The purpose of this study was to investigate changes in individual health care utilisation following choice and privatisation in Swedish primary care from an equity perspective, in subgroups defined by age, gender and family income. In Sweden, equitable access to health care is ensured by law [17] and primary care is vital to enforce this objective. More specifically, we investigated health utilisation in different subgroups before and after introduction of reform in the Skåne Regional Council with 1.2 million inhabitants. In this region, choice of provider and freedom of establishment for private primary care providers was introduced in 2009. Payments to providers are based on capitation payment adjusted for individuals’ diagnosis and socioeconomic indicators, combined with a 3% pay-for-performance scheme. In contrast to most other county councils, there is no payment per visit for registered individuals. A total of 23 new private practices were established following reform, an increase by 17 per cent [18].

## Methods

This paper is based on linked register data from the Skåne Regional Council (individual health care utilisation data) and Statistics Sweden (individual age, gender and family income).

### Population

The study population consisted of all inhabitants 25 to 84 years of age who lived in the Region of Skåne in the year of 2011 and had done so since 2007. The choice of age-span was chosen to include working as well as retired population.

### Variables

All registered individual visits to publicly funded physicians due to health care were included, i.e. repeated measures within individuals. Visits due to preventive care, i.e. mainly child and maternity health care, were excluded. Visits two years before and after the reform were summarized (2007 + 2008 and 2010 + 2011 respectively). The outcome variables were then categorised and dichotomised (i.e., yes versus no) by the type of provider consulted before and after choice was introduced. In this analysis visit to general practitioners was used as the outcome variable.

In the analyses, age was considered as a continuous variable and centred on the mean. Due to the influence of age on income (rising and falling) and health (declining), analyses were made on stratified age-groups, 25–44, 45–64 and 65–84 years of age. Age at the time of the reform (2009) was used.

Each gender was analysed separately.

For our purpose family income was used as a proxy for expected health needs, where individuals with low family income were regarded as individuals with higher health care needs. Low income individuals were those with a pre-tax family income less than the median income for the specific age group and vice versa for high income individuals. Pre-tax personal income included earnings from employment and business, and income transfers (e.g., pension payments, unemployment benefits, or paid sick leave) but not capital returns. Family income in the year of the reform was used. Family income was not equalised regarding family size.

A dichotomous variable was constructed, expressing before or after introduction of choice of provider.

### Statistical method

To analyse the probability of a visit to a general practitioner due to the reform of choice, three logistic regression models were constructed, with visit to a general practitioner (yes or no) as the dependent variable. In the first model (A) the probability was only a function of the time of visit (after or before the reform). In the second model (B) family income (above or below the median) was included. Finally, in the third model (C) the interaction between before-after and family income was included. In all models age was an independent variable. To account for the repeated measures within individuals, we applied multilevel logistic regression [19]. On the first level were visits and on the second level individuals. We estimated only fixed effects, but with this approach we balanced the repeated measures within individuals and the variance on the second level can be interpreted as an expression of how much the individual is affected by the reform. The higher the value of variance is, the lower the impact of the reform is.

The results are presented as odds ratios (OR) with 95% confidence interval. The predicted probability of a visit was calculated from the OR-results and presented as per cent in the figures (age not included in figures). The intra-subject variance (v) is transformed to per cent with the formula (v/(v + 3.29)*100).

All analyses were made with MlWin 2.24 [20].

### Ethics

The study was approved by The Regional Ethical Review Board in Lund, Dnr 2009/547.

## Results

### Descriptive

The study population consisted of 828 988 inhabitants, of these 50.4% were women, see Table 1 for age-groups and ages.

**Table 1:** Number and change of visits and visitors to GP; total and according to family income

	**Age mean**		**Visits before**	**Visits after**	**Visitors before**	**Visitors after**	**Change visits**	**Change visitors**
			**(2 years)**	**(2 years)**			**n (%)**	**n (%)**
**Men 25-44**	34.7	n						
Visits to GP-all		166365	237432	261847	91484	95276	24415 (10.3)	3792 (4.1)
Income below median		85643	113878	126929	43402	45621	13051 (11.5)	2219 (5.1)
Income above median		80722	123554	134918	48082	49655	11364 (9.2)	1573 (3.3)
**Men 45-64**	54.4							
Visits to GP-all		154444	336028	378838	100717	108068	42810 (12.7)	7351 (7.3)
Income below median		75384	177655	195310	49224	52150	17655 (9.9)	2926 (5.9)
Income above median		79060	158373	183528	51493	55918	25155 (15.9)	4425 (8.6)
**Men 65-84**	72.6							
Visits to GP-all		90190	309857	340047	73379	82331	30190 (9.7)	8952 (12.2)
Income below median		45090	160781	171075	36641	36802	10294 (6.4)	161 (0.4)
Income above median		45100	149076	168972	36738	38450	19896 (13.3)	1712 (4.7)
**Women 25-44**	34.7							
Visits to GP-all		160983	376175	419338	110827	114475	43163 (11.5)	3648 (3.3)
Income below median		78160	175822	197920	51151	53139	22098 (12.6)	1988 (3.9)
Income above median		82823	200353	221418	59676	61336	21065 (10.5)	1660 (2.8)
**Women 45-64**	54.5							
Visits to GP-all		153401	470358	517804	115657	121277	47446 (10.1)	5602 (4.9)
Income below median		78558	265618	288359	60308	62654	22741 (8.6)	2346 (3.9)
Income above median		74843	204740	229445	55367	58623	24705 (12.1)	3256 (5.9)
**Women 65-84**	73.3							
Visits to GP-all		103605	432104	468395	88908	90010	36291 (8.4)	1102 (1.2)
Income below median		51788	225746	238731	44685	44567	12985 (5.8)	−118 (−0.3)
Income above median		51817	206358	229664	44223	45443	23306 (11.3)	1220 (2.8)

### Visits to general practitioner (GP)

The inhabitants made a total of 2 161 954 visits to a GP during the years before and a total of 2 386 269 visits after choice was introduced, an absolute rise of 224 315 *visits* (in relative terms 10.4%). The number of *visitors* also increased (n = 30465), in relative terms a rise of 5.2%.

The average number of visits per individual rose from 2.6 to 2.9 or by 10.4%.

### Gender

The relative rise in number of *visits* was 9.9% for women and 11.0% for men. The number of *visitors* also increased with 3.3% for women and 7.6% for men.

The average number of visits per individual rose with 9.9% for women and 11.0% for men.

### Age and gender

The highest absolute and relative rise for men was found in the age-group 45–64 years (n = 42810; 12.7%). For women, the highest absolute rise was found in the age-group 45–64 years (n = 47446) and the highest relative rise in the age-group 25–44 years (11.5%) (Table 1).

The rise in average number of visits was highest for both women and men in the age-group 65–84 years (0.35 and 0.33 respectively). Men had the highest relative rise in average number of visits in the age-group 45–64 (12.7%) and women in the youngest age-group (11.5%) (Table 2).

**Table 2:** Average number of visits

	**Average number of visits**	**Average number of visits**	**Change average number**	**Change average number**
	**before (2 years)**	**after (2 years)**	**of visits**	**of visits n (%)**
**Men 25-44**				
Visits to GP-all	1.43	1.57	0.15	10.3
Income below median	1.33	1.48	0.15	11.5
Income above median	1.53	1.67	0.14	9.2
**Men 45-64**				
Visits to GP-all	2.18	2.45	0.28	12.7
Income below median	2.36	2.59	0.23	9.9
Income above median	2.00	2.32	0.32	15.9
**Men 65-84**				
Visits to GP-all	3.44	3.77	0.33	9.7
Income below median	3.57	3.79	0.23	6.4
Income above median	3.31	3.75	0.44	13.3
**Women 25-44**				
Visits to GP-all	2.34	2.60	0.27	11.5
Income below median	2.25	2.53	0.28	12.6
Income above median	2.42	2.67	0.25	10.5
**Women 45-64**				
Visits to GP-all	3.07	3.38	0.31	10.1
Income below median	3.38	3.67	0.29	8.6
Income above median	2.74	3.07	0.33	12.1
**Women 65-84**				
Visits to GP-all	4.17	4.52	0.35	8.4
Income below median	4.36	4.61	0.25	5.8
Income above median	3.98	4.43	0.45	11.3

### Income group

A further description of the different gender and age-groups according to family income revealed other differences, see Table 1. For both women and men the highest absolute and relative raise in number of visits as well as number of new visitors was found for high income individuals 45–64 years of age. For women there was an almost equal relative rise in visits for low income individuals in the age-group 25–44 years and high income individuals 45–64 years of age (Table 1). The highest rise in the average number of visits was in the oldest age group for both genders with high income (women 0.45 visits and men 0.44 visits). The relative rise in average number of visits was highest for women in the youngest age-group with low income (12.6%) and for men with high income in the age-group 45–64 (15.9%) (Table 2).

### Multilevel logistic regression

**Figure 1:**
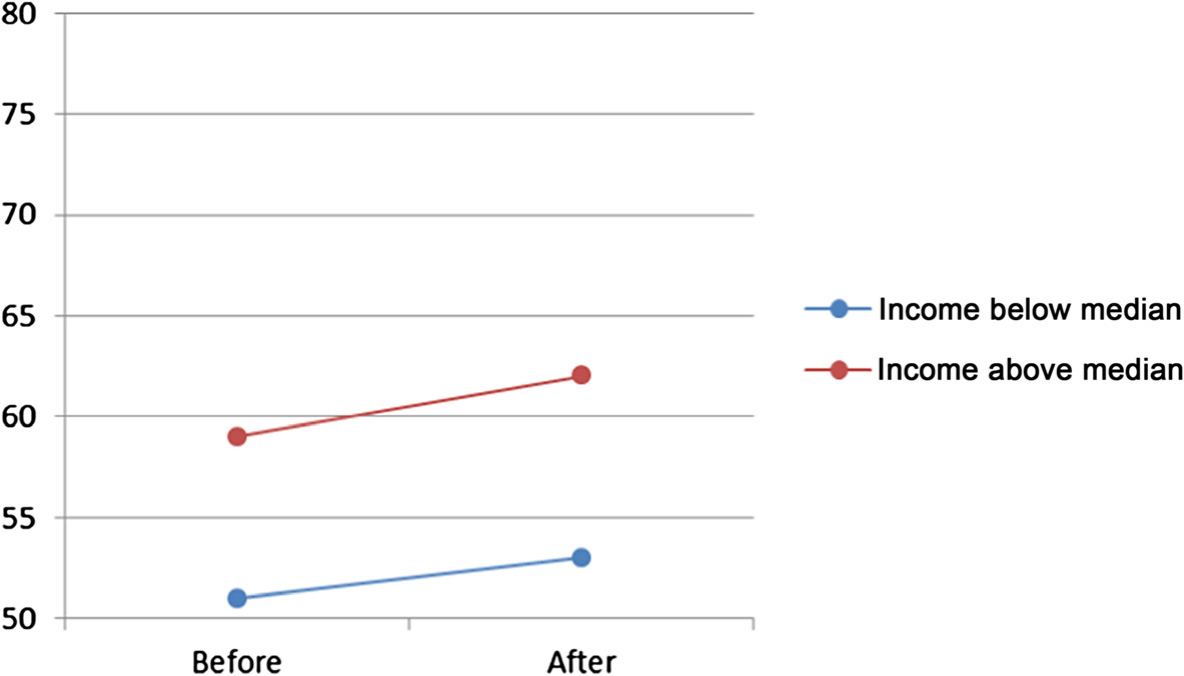
**Predicted probability in per cent (dots) of visit to general practitioner before and after reform, men 25–44, grouped according to family income.** The slope of the connecting lines illustrates the magnitude of change.

**Figure 2:**
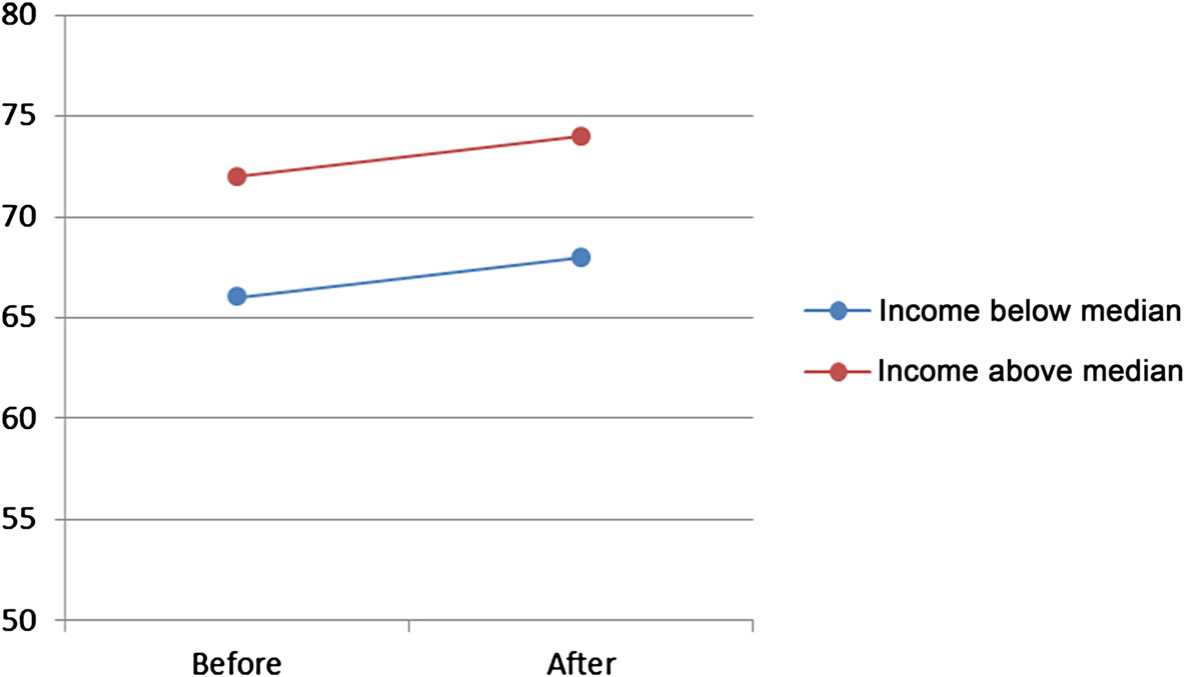
**Predicted probability in per cent (dots) of visit to general practitioner before and after reform, women 25–44, grouped according to family income.** The slope of the connecting lines illustrates the magnitude of change.

**Figure 3:**
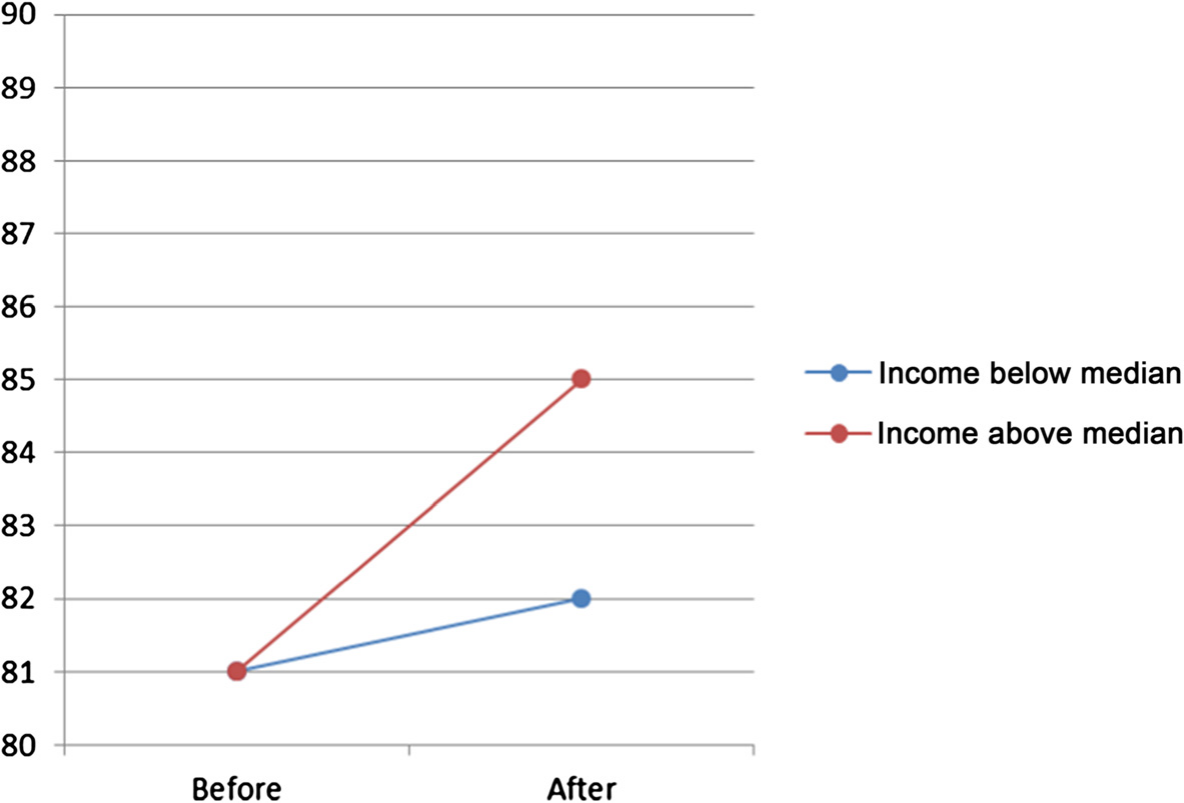
**Predicted probability in per cent (dots) of visit to general practitioner, men 65–84, grouped according to family income.** Effect of interaction between choice and family income group. The slope of the connecting lines illustrates the magnitude of change.

**Figure 4:**
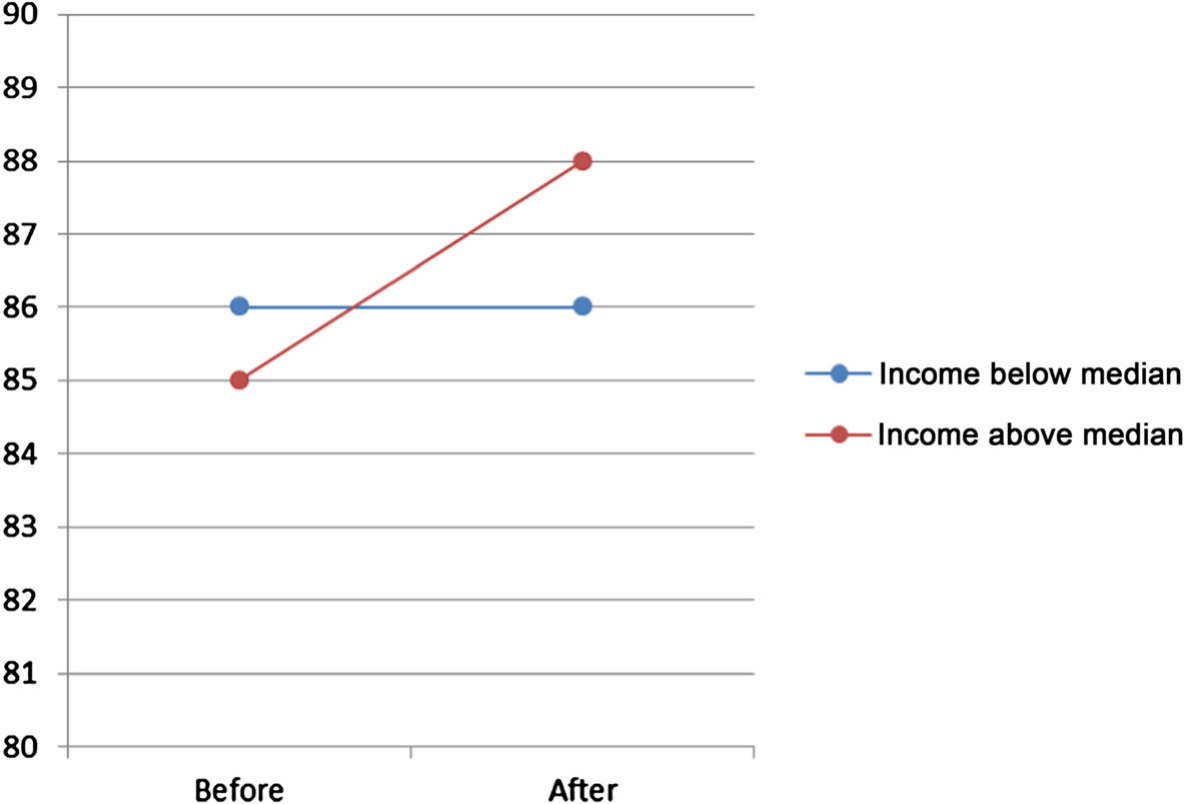
**Predicted probability in per cent (dots) of visit to general practitioner, women 65–84, grouped according to family income.** Effect of interaction between choice and family income group. The slope of the connecting lines illustrates the magnitude of change.

Analyses showed a single effect (model A) on visits to general practitioner of choice of provider, for both women and men, varying from OR 1.10 (women 65–84) to OR 1.24 (men 45–64) (Table 3). However, when socioeconomic status in the form of family income below or above the median income was introduced (model B), the effect was different for different age-groups and gender, Table 3 and Figures 1 and 2. There was a higher probability of visit for men with high family income, especially in the lowest and highest age groups. For women the probability showed the same pattern, except for the age group 45–64 where the probability was lower for women in the high income group. When the interaction between choice and family income group was introduced (model C) the effect was substantial only in the highest age group, both for men and women, Table 3 and Figures 3 and 4.

**Table 3:** Odds ratio for visits to general practitioner

	** Model A**	** Model B**	** Model C**
**Men 25-44**	** OR (95%-CI)**	** OR (95%-CI)**	** OR (95%-CI)**
Choice (after vs. before)	1.10 (1.08-1.11)	1.10 (1.08-1.11)	1.11 (1.09-1.13)
Family income (high vs. low)		1.42 (1.40-1.44)	1.35 (1.31-1.38)
Interaction (choice*family income)			0.98 (0.95-1.01)
Age	1.03 (1.03-1.03)	1.02 (1.02-1.02)	1.02 (1.02-1.02)
Variance (%)	14	14	14
**Men 45-64**			
Choice (after vs. before)	1.25 (1.23-1.27)	1.25 (1.23-1.27)	1.19 (1.17-1.22)
Family income (high vs. low)		1.04 (1.02-1.06)	1.00 (0.98-1.03)
Interaction (choice*family income)			1.04 (1.04-1.04)
Age	1.04 (1.04-1.04)	1.04 (1.04-1.04)	1.04 (1.04-1.04)
Variance (%)	18	18	18
**Men 65-84**			
Choice (after vs. before)	1.15 (1.13-1.18)	1.16 (1.13-1.18)	1.02 (0.99-1.06)
Family income (high vs. low)		1.30 (1.27-1.34)	1.16 (1.11-1.20)
Interaction (choice*family income)			1.29 (1.23-1.35)
Age	1.04 (1.04-1.04)	1.05 (1.04-1.05)	1.05 (1.04-1.05)
Variance (%)	23	23	23
**Women 25-44**			
Choice (after vs. before)	1.11 (1.10-1.13)	1.12 (1.10-1.13)	1.12 (1.10-1.15)
Family income (high vs. low)		1.26 (1.24-1.28)	1.28 (1.24-1.30)
Interaction (choice*family income)			0.99 (0.96-1.02)
Age	1.03 (1.02-1.03)	1.02 (1.02-1.02)	1.02 (1.02-1.02)
Variance (%)	17	16	16
**Women 45-64**			
Choice (after vs. before)	1.23 (1.21-1.25)	1.23 (1.21-1.25)	1.19 (1.17-1.23)
Family income (high vs. low)		0.91 (0.89-0.93)	0.88 (0.86-0.91)
Interaction (choice*family income)			1.07 (1.03-1.10)
Age	1.03 (1.03-1.03)	1.03 (1.03-1.03)	1.03 (1.03-1.03)
Variance (%)	21	21	21
**Women 65-84**			
Choice (after vs. before)	1.10 (1.08-1.12)	1.10 (1.07-1.12)	1.02 (0.98-1.06)
Family income (high vs. low)		1.16 (1.13-1.20)	1.04 (1.00-1.09)
Interaction (choice*family income)			1.25 (1.19-1.32)
Age	1.03 (1.03-1.03)	1.04 (1.03-1.04)	1.04 (1.03-1.04)
Variance (%)	28	28	28

OR = odds ratio.

CI = confidence interval.

Model A = before-after reform.

Model B = A + Family income.

Model C = B + interaction family income and before-after.

Variance on second level (individuals).

The individual variance, ranging from 17 to 28%, indicates a relative consistency in behaviour.

## Discussion

We have explored changes in the individual probability of a visit to a general practitioner before and after the introduction of a reform involving choice and establishment of more private primary care providers in the Skåne Regional Council. Our findings show that the probability increased in all subgroups, i.e. irrespective of age, gender or income. However, for individuals with a family income above median, in both genders and in both absolute and relative numbers, the increase was most pronounced in the age group 45–64. The effect of interaction between family income above median and choice, i.e. who benefitted most from choice of provider, was highest in the elderly, irrespective of gender. This suggests that improved access to primary care following reform in the Region of Skåne has not been equal in relation to socioeconomic status. The rise in absolute numbers of new visitors was highest in men, 45–64 years of age, 30% higher than women of the same age.

Previous studies have shown an unequal utilisation of health care in Sweden [21-23]. In part, this may be attributed to suboptimal investment levels in primary care and relatively few family physicians per capita in comparison with other OECD countries [24]. A previous comparison across OECD countries has shown that primary care generally tends to be distributed with a pro-poor bias, whereas a stronger pro-rich bias is generally present for specialist care [25]. One suggested approach to strengthen equitable access to health care is to introduce patient choice of provider, together with freedom of establishment and privatisation of providers.

Theoretically, it is possible that individuals with higher family income in our study have higher needs, and that introduction of reform therefore has improved equity in terms of utilisation. However, this is a most unlikely explanation, since there is a clear relationship between higher income and better health [26-29]. The difference in the absolute rise in numbers of visitors between men and women in the age group 45–64 is therefore hard to explain with need, as the rise in men was predominantly found in the high income group. There are several more plausible explanations behind the distribution of benefits following reform. One explanation resides in the term of health literacy, i.e. the degree to which individuals can access, process, understand and communicate health-related information that is needed to make informed health decisions [30]. In our context differences in health literacy have an impact on the individual’s ability to capitalize on a new structure in health care focusing on choice of providers. With a higher capacity to make use of this option, larger benefits in terms of increased access may follow. Although low family income is not equal to low health literacy the two concepts do correlate [4,31].

Another explanation is that new private providers following reform favour establishment in areas with income levels above the median. Indeed, both private and public primary care providers in Skåne are paid according to the same principles with capitation adjusted for both illness and socioeconomic status of registered inhabitants. If a private provider chooses to set up a practice in a socioeconomic poor area, payment per registered individual will increase as determined by a care-need-index (CNI) composed by socioeconomic indicators [32]. However, this policy seems to have had limited effect on actual decisions on where to set up new practices. Our own calculations based on register data showed that average care-need-index for the 23 new private practices in September 2010 was 0.96 (median 0.96), compared to an average of 1.08 (median 1.02) for all primary care practices in Skåne. Only 3 out of 22 primary care providers with the highest care-need-index in 2011 (i.e. located in areas with worst socioeconomic conditions) were private [33].

The strength of our study is that we have studied a constant population during five years. This means that no exits or entries have occurred in the studied population, minimizing the external effects of new or missing individuals. In this way we have been able to study the effect of choice of provider in a constant setting. Furthermore, the use of family income more accurately reflects the individuals’ socioeconomic status than individual income. Also, the use of multilevel, logistic regression diminishes the problem of repeated measures. A conscious limitation of this study is the use of dichotomised income. This reduces the variability but our primary aim was to study if there was any effect at all of choice and if income had any influence. A separate analysis was made with income quartiles (not presented here), which confirmed our results. We used family income as a proxy for need. Education as a proxy for need was not possible in our study. Due to the age span we selected, the information on educational level was sparse in people 65 years or older. A further limitation is that our analysis focuses on utilization of GP-visits in primary care. Utilization of outpatient specialist care and visits to other health care staff has an impact on the distribution of total benefits.

The study is based on register information that has been gathered for administrative purposes. Sweden has a long tradition of keeping population and health care registers, and well-developed systems are in place for recording, storing, and managing information. The database we studied has been checked for registration errors. Family income are not self-reported, but are based on official statistics maintained by Swedish authorities, increasing the validity of data. Information regarding visits to physicians is recorded by the county patient administrative system that covers all the health care facilities; the dependent variable used in our study (i.e. visits to a GP) has a high validity. The use of family income as an index of socioeconomic status and thus a proxy for health care need can be discussed in terms of validity. However, in the absence of self-rated health, this is a good indicator of health [28].

## Conclusions

Reforms involving choice for individuals and freedom of establishment for private providers in the Swedish Region of Skåne have improved access to GP-visits generally irrespective of age, gender and income. However, for individuals with a family income above the median, in both genders and ages above 64, the increase was more pronounced. This suggests that improved access to primary care following reform has not been equal in relation to socioeconomic status.

There are several plausible explanations behind the unequal distribution of benefits following reform. One explanation resides in the term of health literacy and to which extent individuals are able to capitalize on a new structure in health care focusing on choice of providers. Another explanation is that new private providers after the reform more often have established themselves in areas with favourable socioeconomic conditions. Both private and public primary care providers are paid according to the same principles, with capitation adjusted for both illness and socioeconomic status, but this policy seems to have had limited effect on actual decisions on where to set up new practices.

## Competing interests

The authors declare that they have no competing interests.

## Authors’ contributions

AB collected and analysed all data. AB and AA drafted, read and approved the final manuscript together.

## Pre-publication history

The pre-publication history for this paper can be accessed here:

http://www.biomedcentral.com/1472-6963/13/452/prepub
